# Neutralizing antibodies against SARS-CoV-2 virus after vaccination in patients with neurofibromatosis type 1

**DOI:** 10.1038/s41392-023-01498-1

**Published:** 2023-06-02

**Authors:** Qiao-ling Ruan, Zhi-chao Wang, Cheng-jiang Wei, Wei Wang, Qing-luan Yang, Jing Wu, Yan-min Wan, Ling-ling Ge, Wen-hong Zhang, Qing-feng Li

**Affiliations:** 1 Department of Infectious Diseases, National Medical Center for Infectious Diseases, Shanghai Key Laboratory of Infectious Diseases and Biosafety Emergency Response, Huashan Hospital, Fudan University, Shanghai, People’s Republic of China; 2 Department of Plastic and Reconstructive Surgery, Shanghai Ninth People’s Hospital, Shanghai Jiao Tong University School of Medicine, Shanghai, People’s Republic of China; 3 Shanghai Huashen Institute of Microbes and Infections, Shanghai, People’s Republic of China

**Keywords:** Neurodevelopmental disorders, Vaccines

**Dear Editor**,

The severe acute respiratory syndrome coronavirus 2 (SARS-CoV-2) has triggered a COVID-19 pandemic that has caused high morbidity and mortality worldwide.^1^ COVID-19 vaccines are urgently needed to protect all susceptible populations from SARS-CoV-2 infection. Neurofibromatosis type 1 (NF1) is a hereditary dominant disease that affects approximately one in every 3000 newborns.^2^ Due to its rarity, no data currently exists on the efficacy and safety of COVID-19 vaccinations for patients with NF1. This concern is frequently raised by the patient community in clinical practice at China’s largest NF1 center. The primary issue is the unclear safety and efficacy profile. In the present study, we evaluated the neutralizing antibody responses generated by SARS-CoV-2 whole-virion inactivated vaccines in NF1 patients.

Between April 15 and September 15, 2021, a total of 32 patients with NF1 who had no previous infection with SARS-CoV-2 were prospectively enrolled in this study. Age, sex, body mass index (BMI), and *Nf1* gene mutation information were obtained for the patients (Supplementary Tables S1–3). All patients provided written informed consent. Each participant was administered two doses of SARS-CoV-2 whole-virion inactivated vaccines (CoronaVac or BBIBP-CorV) with a 4-week interval between doses, adhering to the guidelines for SARS-CoV-2 vaccination set by the National Health Commission of China. The collection of adverse events (AEs) induced by vaccines, including injection-site and systemic symptoms, was conducted through the implementation of a standardized questionnaire. Plasma samples were procured at enrollment and 14 days post second vaccine dose to evaluate for anti-SARS-CoV-2 responses. Plasma surrogate virus neutralization tests were used to quantitatively determine the neutralizing activity of the immunoassay using SuperFlex^TM^ Anti-SARS-CoV-2 Neutralizing Antibody Kit (Suzhou Sym-Bio Lifescience Co., Ltd, China). Total anti-SARS-CoV-2 and immunoglobulin G (IgG) antibodies were measured using SuperFlex^TM^ Anti-SARS-CoV-2 and Anti-SARS-CoV-2 IgG Kit. Antibodies that block angiotensin-converting enzyme 2 (ACE2) binding to the SARS-CoV-2 spike, including the virus variants, were determined by the V-PLEX COVID-19 ACE2 Neutralization Kits (Meso Scale Diagnostics, LLC, U.S.A.).

Two weeks after administration of the second dose, significant increases in total anti-SARS-CoV-2 antibodies and IgG levels, as well as neutralizing antibodies, were observed in patients with NF1. The median antibody levels for total anti-SARS-CoV-2 and IgG were 73.81 binding antibody units (BAU)/mL (interquartile range [IQR] 23.47–196.225) and 79.0 (34.71–164.85) IU/mL, respectively (Fig. 1a, b). The median level of the neutralizing antibodies was 97.81 (IQR 45.83–332.78) IU/mL (Fig. 1c). Comparison with the data from 28 healthy controls (median age: 37.5 years, male: 57.1% [16/28], median BMI: 23.47 kg/m^2^) who received the same vaccine regimen revealed that patients with NF1 had significantly higher immune responses to the vaccine. In healthy controls, the median antibody levels for total anti-SARS-CoV-2 and IgG, as well as neutralizing antibodies, were 6.33 (IQR 1.26–59.0) BAU/mL, 10.59 (IQR 2.68–32.39) BAU/mL, and 19.89 (IQR 4.63–63.69) IU/mL, respectively (Fig. 1d–f). The mean titer of neutralizing antibodies in patients with NF1 and controls were 403.25 and 64.96, respectively (Fig. 1g). Subsequently, we assessed the relative impact of each variant of SARS-CoV2. Inhibition ratios of ACE2 binding to variants of SARS-CoV-2 B.1.1.7 (Alpha) (52.33, IQR [40.43, 67.42]), B.1.351 (Beta) (27.59, IQR [19.94, 51.90]), and P.1 (Gamma) (27.85, IQR [13.58, 50.40] were significantly decreased in patients compared with the original SARS-CoV-2 strain WA1/2020 (64.10, IQR [48.94, 82.06]) (Fig. 1h). Furthermore, we conducted a rapid multiplex cytokine assay to quantify a panel of the interleukins (including IL-1β, IL-2, IL-4, IL-6, IL-8, IL-10, IL-12p70, and IL-13), along with IFN-γ and TNF-α, at baseline and 14 days post the second dose. In patients with NF1, we observed statistically significant increases in IL-1β (*P* < 0.001) and IL-8 (*P* = 0.017) following vaccination, as compared to the controls (Supplementary Fig. S1). IL-1β and IL-8, both potent pro-inflammatory cytokines, are known to be pivotal in modulating the immune response. Notably, 25 out of 27 patients with NF1 were infected with the Omicron variant between December 2022 and January 2023, with no severe cases.

**Fig. 1:**
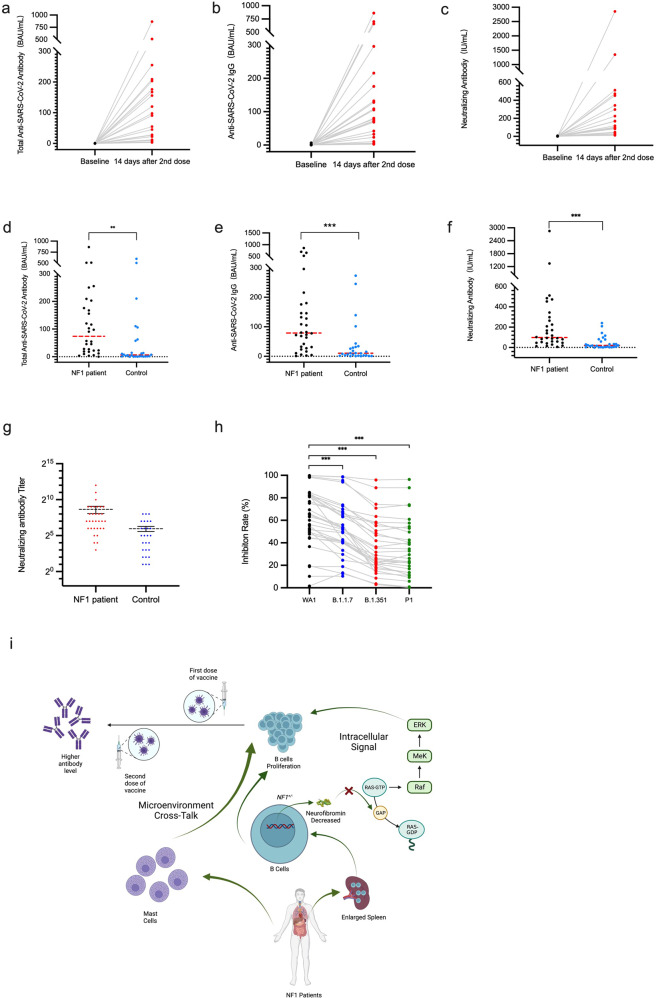
Immune responses of the patients with NF1 to whole-virion inactivated SARS-CoV-2 vaccines. **a**–**c** Total anti-SARS-CoV and IgG, as well as neutralizing antibodies at baseline and 14 days, post the second dose in patients (*N* = 22). **d**–**f** Total anti-SARS-CoV and IgG, as well as neutralizing antibodies, were compared between patients (*N* = 32) and healthy controls (*N* = 28). **P < 0.01,***P < 0.001. **g** Neutralizing antibodies titer of patients (*N* = 32) and healthy controls (*N* = 28). **h** The inhibition ratios of ACE2 binding to the SARS-CoV-2 spike of variants WA1/2020, B.1.1.7 (Alpha), B.1.351 (Beta), and P.1 (Gamma). **i** A potential mechanism for the significantly activated immune system observed in patients in response to COVID-19 vaccinations (illustrated using BioRender.com)

Regarding safety concerns, 14 (43.75%) participants reported AEs within 3 days following vaccination, encompassing both injection-site and systemic reactions. The prevailing AE was injection-site reactions (eight patients [25.0%]). Other systemic AEs included fatigue (six patients [18.75%]), headache (four patients [12.50%]), dizziness (three patients [9.38%]), diarrhea (three patients [9.38%]), and cough (one patient [3.12%]).

The present study has limitations that need to be acknowledged. First, the sample size was restricted owing to the rarity of NF1, which precluded the possibility of conducting additional subgroup analyses.^3^ Second, further validation studies are required in patients with NF1 from diverse ethnic backgrounds and in the elderly age group.

In conclusion, patients with NF1 will benefit from a COVID-19 vaccination with acceptable safety and increased efficacy. This is encouraging news for the patient population, not only as it represents the first evidence of the efficacy of COVID-19 vaccination in NF1 patients but also in light of the global shortage and the unequal distribution of vaccines. Although the promising results are from a small cohort, the findings suggest that the immune cells of patients with NF1 were activated in response to the COVID-19 vaccination, consistent with unimpaired lymphocyte development in the absence of neurofibromin.^4^ Previous transgenic models of B and T cells lacking the expression of neurofibromin led to the increased proliferation of immune cells.^4,5^ The underlying mechanisms might be the hyperactivation of the RAS signaling pathway, and the crosstalk between mast and B cells might also be essential in B-cell development^5^ (Fig. 1i), highlighting a need for further investigation. Patients with NF1 should be less hesitant to receive COVID-19 vaccinations, and it appears that two doses of the vaccine could provide satisfactory protection in terms of neutralizing antibodies. Therefore, strategies for completing a two-dose vaccination regimen should be prioritized in the NF1 community.

## Supplementary information

Supplementary material revision.pdf

## Data Availability

All data presented in this study are available on request.

## Materials availability

All materials presented in this study are available on request.
