# Supplementary material for: Neutralizing antibodies against SARS-CoV-2 virus after vaccination in patients with neurofibromatosis type 1
Source: Signal Transduct Target Ther. 2023 Jun 2;8:233. doi: 10.1038/s41392-023-01498-1 (PMC10236388; doi:10.1038/s41392-023-01498-1)
Supplement: Supplementary file 1 — Supplementary material revision.pdf [file 41392_2023_1498_MOESM1_ESM.pdf]

Supplementary Materials for

SARS-CoV-2 Virus after Vaccination in Patients with Neurofibromatosis Type 1

Qiao-ling Ruan[#1], Zhi-chao Wang[#2], Cheng-Jiang Wei[#2], Wei Wang[#2], Qing-luan Yang[#1], Jing Wu[1], Yan-min Wan[1], Ling-ling Ge[2], Wen-hong Zhang[1,3], Qing-feng Li[2].

[1] Department of Infectious Diseases, National Medical Center for Infectious Diseases, Shanghai Key Laboratory of Infectious Diseases and Biosafety Emergency Response, Huashan Hospital, Fudan University, Shanghai, People's Republic of China

[2] Department of Plastic and Reconstructive Surgery, Shanghai Ninth People's Hospital, Shanghai Jiao Tong University School of Medicine, Shanghai, People's Republic of China

[3] Shanghai Huashen Institute of Microbes and Infections, People's Republic of China

[#]These authors contributed equally to this work and are considered as the first authors.

Corresponding to:

Prof. Qing-feng Li, MD, PhD, & Prof. Wen-hong Zhang, MD, PhD

dr.liqingfeng@shsmu.edu.cn & zhangwenhong@fudan.edu.cn

This PDF file includes:

Materials and Methods

Supplementary Figure S1

Supplementary Table S1 to S3

Materials and Methods

Study population

The largest neurofibromatosis type 1 (NF1) center in China invites patients to take the COVID-19 vaccination to prevent infection. Between April 15 and September 15, 2021, 32 patients with NF1 who had not received a COVID-19 vaccination were prospectively enrolled in this study. Meanwhile, 28 healthy individuals who had not received a COVID-19 vaccination were also enrolled as controls. The exclusion criteria were as follows: 1) SARS-CoV-2 infection confirmed by positive reverse transcription-polymerase-chain reaction assay; 2) A history of infection with SARS-CoV-2, or a history of contact with cases of confirmed or suspected SARS-CoV-2 infection; 3) Presence of fever, cough, runny nose, sore throat, diarrhea, dyspnea, or tachypnoea within 7 days before the screening visit; 4) Allergy to any ingredient included in SARS-CoV-2 vaccines or a history of severe allergy; 5) Pregnant; 6) Suffering from severe liver or kidney disease, uncontrollable hypertension, malignant tumor, or other acute diseases or chronic diseases with acute exacerbation; and 7) Known immunosuppressive or immunodeficient state, including human immunodeficiency virus infection, and has been prescribed systemic immunosuppressants within 3 months prior to the day of screening. Baseline characteristics were collected, including age, sex, and body mass index, as well as *Nf1* gene mutation information for patients with NF1.

All participants received two doses of an inactivated whole-virion SARS-CoV-2 vaccine (CoronaVac or BBIBP-CorV). The interval between the first and second SARS-CoV-2 vaccine doses was 4 weeks, according to the guidance for the SARS-CoV-2 vaccination by the National Health Commission of the People's Republic of China.

The study was approved by the Ethics Committee from Shanghai Ninth People’s Hospital, Shanghai Jiao Tong University (SH9H-2019-T163-2), and Huashan Hospital Shanghai Medical College Fudan University (2021M-002). Written informed consent was obtained from all enrolled patients.

Safety assessment

Side effects of the vaccines, including injection-site symptoms and systemic symptoms, were collected using a standard questionnaire.

Plasma surrogate virus neutralization, total anti-SARS-CoV-2 antibody, and immunoglobulin G tests

Plasma samples of the enrolled subjects were taken at enrollment and 14 days after the second dose. The plasma surrogate virus neutralization test was used to quantitatively detect neutralizing antibodies to SARS-CoV-2 by immunoassay using the SuperFlex Anti-SARS-CoV-2 Neutralizing Ab Kit (Suzhou Sym-Bio Lifescience Co., Ltd, China) according to the manufacturer’s instructions. The authors used superparamagnetic microparticles in combination with direct chemiluminescence technology to detect antibodies in the plasma samples. Plasma was serial diluted before detection, and 50 μl of the diluted sample was added to the sample wells. Then, 50 ul of SARS-CoV-2 receptor binding domain protein labeled with acridinium ester was added. Magnetic particles coated with human angiotensin-converting enzyme 2 (ACE2) protein were then added to form a competitive model. The unbound substance was removed by washing, and the luminescence value of the chemiluminescence reaction was measured using pre-trigger and trigger solutions. Signals were captured using the PerkinElmer SuperFlex automatic chemiluminescence immunoassay analyzer.

The total amount of anti-SARS-CoV-2 antibodies was measured using a SuperFlex Anti-SARS-CoV-2 Ab Kit (Suzhou Sym-Bio Lifescience Co., Ltd, China). A sample was added to a sample well and then bound with the magnetic particles coated with SARS-CoV-2 antigens. An immunocomplex was then formed together with a biotinylated SARS-CoV-2 antigen. After washing, an acridinium ester-labeled anti-biotin antibody was added to form a new immunocomplex. Unbound substances were removed by washing, and the luminescence value of the chemiluminescence reaction was measured using pre-trigger and trigger solutions.

The anti-SARS-CoV-2 immunoglobulin G (IgG) was measured using a SuperFlex Anti-SARS-CoV-2 IgG Kit (Suzhou Sym-Bio Lifescience Co., Ltd, China). A specimen was added to the specimen well and then bound with magnetic particles coated with SARS-CoV-2 antigens. After washing, an acridinium ester labeled anti-human IgG antibody was added to form an immunocomplex. Unbound substances were removed by washing, and the luminescence value of the chemiluminescence reaction was measured using pre-trigger and trigger solutions.

The antibody assay was analyzed using the original scale, and the results were then converted to the World Health Organization's international standard units using the conversion factors supplied by the laboratory.

Inhibition ratios of angiotensin-converting enzyme 2 binding

Antibodies that block the binding of ACE2 to the SARS-CoV-2 spike, including variants of the SARS-CoV-2 virus, were measured using V-PLEX COVID-19 ACE2 Neutralization Kits (Meso Scale Diagnostics, LLC, U.S.A.).

Cytokine assay

A rapid multiplex cytokine assay was used to measure the interleukins (including IL-1β, IL-2, IL-4, IL-6, IL-8, IL-10, IL-12p70, and IL-13), IFN-γ, and TNF-α at the baseline and 14 days following the second dose. The V-PLEX Proinflammatory Panel 1 Human Kit from Meso Scale Discovery was used according to the manufacturer's protocol. Plates were read on a MESO SECTOR S 600 imager and analyzed using the Discovery Workbench software.

Statistical analysis

The summary statistics for individuals were shown as the median with interquartile ranges. Post-vaccination side effects were presented as numbers and proportions. The Mann–Whitney U test was used for the continuous variables to assess the statistical significance between the groups. The Wilcoxon matched-pairs signed rank test was used to compare the inhibition ratios between WA1/2020 and the variants. *P* values of less than 0.05 were considered significant. Graphpad Prism (version 9.1) was used for the statistical analysis.

Supplementary Figure and Figure legend

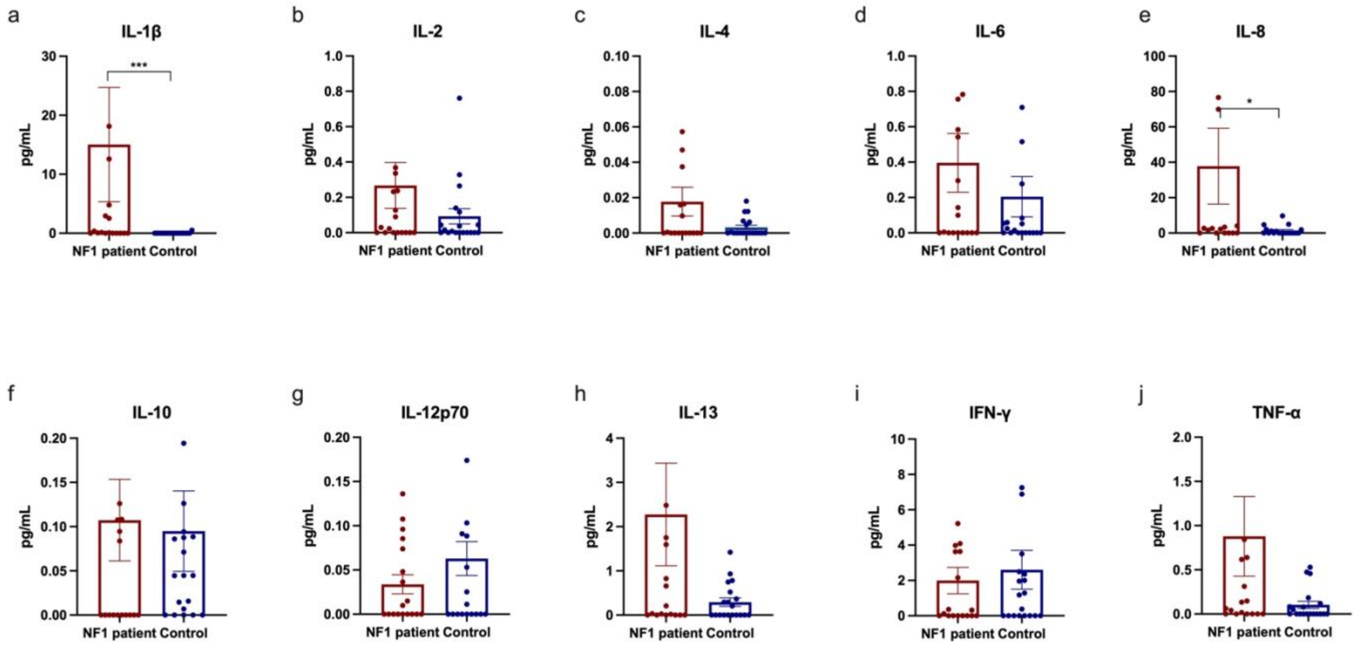

Supplementary Figure S1. The electrochemiluminescence immunoassay of the plasma sample from patients with NF1 and the controls. The results are presented as the cytokines concentration at 14 days after the second vaccination minus the cytokines concentration at the baseline. (a) IL-1β, (b) IL-2, (c) IL-4, (d) IL-6, (e) IL-8, (f) IL-10, (g) IL-12p70, (h) IL-13, (i) IFN-γ, (j) TNF-α. Data are expressed as ±SEM (N=18 in the patient with NF1 group, N=19 in the control group; *$P<0.5$, ***$P<0.001$).

Supplementary Table S1. Genetic tests of *NF1* gene in all enrolled patients.

Patient ID	Mutation Gene	Chromosome	Exon	Mutation site	Mutation type	Target protein	NIH NF1 clinical diagnosis Criteria
NF1	*NF1*	chr17:29550585	exon16	c.1845G>C	Heterozygote	p.K615N（p.Lys615Asn）	Yes
NF2	*NF1*	chr17:29508726	exon7	c.655-2A>G	Heterozygote	splicing	Yes
NF3	*NF1*	chr17:29557401	exon23	c,311+1G>A	Heterozygote	splicing	Yes
NF4	*NF1*	chr17:29486055_29486056	exon3	c.233delA	Heterozygote	p.N78Ifs*7	Yes
NF5	*NF1*	chr17:g.29654664C>T	exon37	c.5353C>T	Heterozygote	p.Gln1785Ter	Yes
NF6	No report						Yes
NF9	No report						Yes
NF8	*NF1*	chr17:29665753_29665757	exon45	c.6789_6792delTTAC	Heterozygote	p.Y2264Tfs*5	Yes
NF19	*NF1*	chr17:29653154-29653154	exon36	c.5088_5089dupGC	Heterozygote	p.H1697Rfs*3	Yes
NF11	No report						Yes
NF12	No report						Yes
NF16	*NF1*	chr17:29486055_29486056	exon3	c.233delA	Heterozygote	p.N78Ifs*7	Yes
NF17	*NF1*	chr17:29486055_29486056	exon3	c.233delA	Heterozygote	p.N78Ifs*7	Yes
NF20	*NF1*	chr17:29508775_29508775	exon3	c.702G>A	Heterozygote	p.Leu234=	Yes
NF21					Heterozygote		Yes
NF24	*NF1*	chr17:29528055_29528057	exon10	c.1064_1065delAC	Heterozygote	p.N355Tfs*4	Yes
NF28	No report						Yes
NF30	No report						Yes
NF31	No report						Yes
NF39	*NF1*	chr17:29509648_29509648	exon8	c.852_853dupAT	Heterozygote	p.S285Yfs*11	Yes
NF51					Heterozygote		Yes
NF10	*NF1*	chr17（GRCH37）		c.202dupA	Heterozygote	p.Met68Asnfs*9	Yes
NF25	No report						Yes
NF47	*NF1*	/	/	/	Heterozygote	/	Yes
NF32	*NF1*	chr17:29560048_29560049	exon3	c.3525_2536del	Heterozygote	p.Arg1176Serfs*18	Yes
NF34	*NF1*	chr17:29588786	exon34	c.4572C>G	Heterozygote	p.Y1524X	Yes
NF35	No report						Yes
NF40	No report						Yes
NF43	No report						Yes
NF52	No report						Yes
NF53	*NF1*	chr17:29664443_29664443	exon42	c.6422dupA	Heterozygote	p.Y2141X	Yes
NF54	*NF1*	chr17:29556846-29556850	exon22	c.2851-6_2851-3delCTTT(Splicing)	Heterozygote		Yes

Supplementary Table S2. Demographics and parameters for COVID-19 vaccinations in NF1 patients.

Number	Gender	Age	Height (CM)	Weight （KG)	BMI	Baseline IgG	Baseline Ab	Baseline Neutralizing Ab			14 days IgG	14 days Ab	14 days Neutralizing Ab			MSD Inhibitor rates				ADVERSE EVENTS									
						Concentration (BAU/mL)	Concentration (BAU/mL)	Concentration (IU/mL)	Titer	Inhibition rate	Concentration (BAU/mL)	Concentration (BAU/mL)	Concentration (IU/mL)	Titer	Inhibition rate					Local response	Fatigue	Fever	Shivering	Lost of appetite	Vomit	Dizzy	Headache	Diarrhea	Other
																Spike	B.1.1.7	B.1.351	P1										
NF1	Female	26	166	55	19.96	0.488	0.076	4.801	8	7.34%	175.7	255.1	225.1	256	95.51%	50.49	24.52	20.5	11.25	0	0	0	0	0	0	0	0	0	
NF2	Female	25	160	60	23.44	0.234	0	2.782	4	3.25%	32.34	11.94	28.87	32	52.86%	64.95	52.76	31.71	32.78	0	1	0	0	0	0	0	0	1	
NF3	Male	19	170	69	23.88	0.376	0.041	0	0	0	295.8	167.3	93.51	128	85.37%	49.33	41.1	23.81	22.28	1	0	0	0	0	0	0	0	0	
NF4	Male	61	168	65	23.03	0.319	0	3.355	4	4.34%	23.34	9.731	40.77	64	64.63%	55.13	33.34	18.27	13.42	0	0	0	0	0	0	0	0	1	
NF5	Female	32	155	48.45	20.17	0.234	0.069	2.785	4	3.26%	80.34	54.82	117.3	128	88.94%	36.67	29.78	15.04	9.33	0	0	0	0	0	0	0	0	0	
NF6	Female	36	155	65	27.06	6.64	0.037	2.888	4	3.45%	77.66	203	297.9	512	97.14%	98.23	94.08	74.27	73.11	0	1	0	0	0	0	1	1	0	
NF9	Female	37	160	52	20.31	0.289	0.066	0.94	0	0.45%	128.1	155.9	512.2	1024	99.11%	1.53	13.32	2.97	0.25	0	0	0	0	0	0	0	0	0	
NF8	Female	34	154	63	26.56	0.301	0.067	2.032	4	1.97%	10.37	22.3	46.05	64	68.43%	51.65	49.02	16.89	13.63	1	0	0	0	0	0	0	0	0	
NF19	Male	27	168	64	22.68	0.307	0.004	2.439	4	2.65%	2.579	3.424	12.26	16	24.47%	65.86	53.57	21.89	29.52	0	0	0	0	0	0	0	0	0	
NF11	Female	37	145	50.5	24.02	1.05	0.042	0.903	0	0.41%	5.91	5.351	18.58	32	37.31%	69.36	53.75	26.27	23.14	0	0	0	0	0	0	0	0	0	
NF12	Male	39	172	75	25.35	0.173	0.07	0	0	0	108.5	45.49	91.6	128	85.00%	76.76	67.32	41.91	38.68	0	0	0	0	0	0	0	0	0	
NF16	Female	66	145	45	21.40	0.36	0.011	0	0	0	215.8	175.9	473.1	512	98.89%	99.72	98.56	88.98	88.93	0	1	0	0	0	0	1	0	0	cough
NF17	Male	24	172	115	38.87	0.713	0	0.399	0	0	701	511.3	1347	2048	99.77%	18.88	18.86	8.64	5.84	0	1	0	0	0	0	0	0	0	
NF20	Male	31	170	85	29.41	0.266	0	0	0	0	41.81	97.69	170.6	256	93.28%	75.24	67.71	46.68	42.52	0	0	0	0	0	0	0	0	0	
NF21	Female	25	160	54	21.09	0.351	0.023	4.325	8	6.31%	71.87	209	45.76	64	68.24%	84.83	73.7	50.8	54.43	1	0	0	0	0	0	0	0	0	
NF24	Male	21	166	62	22.50	0.84	0.034	5.314	8	8.47%	73.11	26.99	83.27	128	83.22%	99.8	98.77	95.88	96.32	0	0	0	0	0	0	0	0	0	
NF28	Male	12	160	55	21.48	0.467	0.069	7	8	12.32%	659.8	513.3	452.7	512	98.76%	56.24	49.93	35.5	34.59	0	0	0	0	0	0	0	0	0	
NF30	Male	27	175	56.5	18.45	0.196	0.032	1.86	2	1.70%	4.21	3.47	14.79	16	29.92%	98.86	95.61	70.79	73.9	0	0	0	0	0	0	0	0	0	
NF31	Female	16	158	62	24.84	2.245	0.072	0	0	0	132.3	120.1	167.1	256	93.08%	19.52	12.64	3.67	0.91	0	0	0	0	0	0	0	0	0	
NF39	Female	25	165	70	25.71	0.494	0.029	2.887	4	3.45%	68.8	28.25	102.1	128	86.85%	52.79	41.11	28.9	26.17	0	0	0	0	0	0	0	0	0	
NF51	Female	33	158	52	20.83	0.332	0.37	0	0	0	105.4	90.8	344.4	512	97.61%	82.47	72.74	55.23	52.9	1	0	0	0	0	0	0	0	0	
NF10	Female	22	160	54	21.09	0.388	0.969	0	0	0	861.9	864.7	2853	4096	99.94%	81.99	63.92	57.51	49.57	1	0	0	0	0	0	0	1	0	
NF25	Male	35	180	83	25.62	N.A	N.A	N.A	N.A	N.A	180.2	305.5	264	512	96.50%	10.25	10.34	-0.48	-2.45	0	0	0	0	0	0	0	0	0	
NF47	Female	20	154	56	23.61	N.A	N.A	N.A	N.A	N.A	82.08	45.59	87.24	128	84.11%	81.87	69.95	63.48	60.93	0	0	0	0	0	0	0	0	0	
NF32	Female	33	160	60	23.44	N.A	N.A	N.A	N.A	N.A	145.9	22.92	52.9	64	72.46%	44.6	39.93	21.25	16.91	0	0	0	0	0	0	0	0	0	
NF34	Female	27	145	43	20.45	N.A	N.A	N.A	N.A	N.A	146.1	102.6	208	256	94.94%	84.16	65.79	58.4	57.6	0	0	0	0	0	0	0	0	0	
NF35	Female	26	158	56	22.43	N.A	N.A	N.A	N.A	N.A	63.4	56.81	140.2	256	91.22%	80.94	63.08	48.6	40.37	1	0	0	0	0	0	0	0	0	
NF40	Female	0	165	51	18.73	N.A	N.A	N.A	N.A	N.A	16.3	18.8	59.57	64	75.64%	82.28	42.79	25.29	22.07	1	1	0	0	0	0	0	1	0	
NF43	Female	30	155	58	24.14	N.A	N.A	N.A	N.A	N.A	31.22	116.4	81.54	128	82.80%	60.08	51.89	36.41	39.99	1	0	0	0	0	0	0	0	0	
NF52	Female	34	158	48.1	19.27	N.A	N.A	N.A	N.A	N.A	27.31	17.73	58.81	64	75.31%	47.78	40.6	22.76	19.3	0	0	0	0	0	0	0	0	0	
NF53	Male	13	155	45	18.73	N.A	N.A	N.A	N.A	N.A	529.1	250.1	484.7	512	98.96%	63.24	55.92	12.85	10.98	0	1	0	0	0	0	1	1	1	
NF54	Male	30	165	60	22.04	N.A	N.A	N.A	N.A	N.A	0.905	0.429	6.206	8	10.49%	44.04	41.56	20.87	23.55	0	0	0	0	0	0	0	0	0	

Supplementary Table S3. Demographics and parameters for COVID-19 vaccinations in healthy controls

Number	Number	Gender	Age	Hight	Weight(kg)	BMI	14 days IgG	14 days Ab	14 days Neutralizing Ab		
							Concentration(BAU/mL)	Concentration(BAU/mL)	Concentration（IU/mL）	Titer	Inhibition rate
1	C-wy-002	Male	40	181	103	31.4398	0.745	0.872	3.272	4	13.89%
2	C-lz-003	Male	56	170	80	27.6817	2.193	0.064	1.443	2	6.52%
3	C-njw-004	Male	40	169	70	24.5089	0.547	7.503	0.052	0	0
4	C-sb-005	Male	41	177	90	28.7274	1.693	1.321	5.093	8	20.47%
5	C-ll-007	Male	40	173	98	32.7442	0.902	0.564	1.89	2	8.39%
6	C-lm-008	Male	45	170	80	27.6817	5.638	1.077	7.558	8	28.24%
7	C-wl-009	Male	39	189	86	24.0755	31.73	57.83	78.76	128	85.77%
8	C-ljx-010	Male	29	179	73	22.7833	1.123	1.914	1.846	2	8.20%
9	C-crx-011	Male	30	175	85	27.7551	273.2	210.1	240.9	256	>95%
10	C-xc-012	Male	26	173	73	24.3911	3.09	0.439	2.09	4	9.21%
11	C-pxf-014	Male	34	176	70	22.5981	8.399	7.140	21.9	32	56.32%
12	C-xlh-015	Male	33	168	57	20.1956	139.4	600.0	81.46	128	86.29%
13	C-zy-016	Male	27	170	86	29.7578	245.4	479.8	209.3	256	>95%
14	C-zj-017	Female	50	158	51	20.4294	0.507	0.350	3.056	4	13.05%
15	C-lx-018	Female	36	162	62	23.6244	10.91	10.37	20.69	32	54.71%
16	C-lyh-019	Female	53	165	53	19.4674	10.27	3.297	15.99	16	47.40%
17	C-xt-020	Female	39	160	48	18.75	18.6	5.524	32.7	64	67.29%
18	C-ql-023	Female	37	168	60	21.2585	11.02	13.61	30.12	32	65.12%
19	C-xxl-024	Female	34	160	47	18.3594	29.13	11.62	32.77	64	67.34%
20	C-xyj-025	Female	38	175	83	27.102	25.81	14.07	32.42	64	67.06%
21	C-xyf-026	Female	34	162	58	22.1003	15.32	4.931	19.1	32	52.43%
22	C-wsy-027	Female	27	163	62	23.3355	6.565	1.737	14	16	43.71%
23	C-yss-028	Female	59	156	50	20.5457	2.848	1.090	5.513	8	21.88%
24	C-hm-030	Female	35	164	67	24.9108	101.6	110.5	116.1	128	90.87%
25	C-zs-031	Male	22	176	68	21.9525	43.59	105.9	58.67	64	80.65%
26	C-Wzr-032	Male	35	175	74	24.1633	3.284	1.381	8.406	16	30.64%
27	C-HLk-035	Male	40	170	66	22.8374	34.35	62.60	98.54	128	88.93%
28	C-YW-036	Female	39	163	55	20.7008	498.2	506.4	143.5	256	92.96%
